# The APOE paradox: divergent genetic influences on hemorrhagic stroke risk—A meta-analysis

**DOI:** 10.3389/fstro.2026.1684121

**Published:** 2026-03-04

**Authors:** Manabesh Nath, Astha Rai, Shubham Misra, Pradeep Kumar

**Affiliations:** 1 Department of Neurology, All India Institute of Medical Sciences, New Delhi, India; 2 Department of Endocrinology, All India Institute of Medical Sciences, New Delhi, India; 3 Department of Neurology, Yale University, New Haven, CT, United States; 4 Clinical Research Unit, All India Institute of Medical Sciences, New Delhi, India

**Keywords:** APOE alleles, APOE genotype, Apolipoprotein E, hemorrhagic stroke, intracerebral hemorrhage

## Abstract

**Background:**

Apolipoprotein E (APOE) regulates lipid metabolism and neuronal repair, yet its alleles show contrasting effects on hemorrhagic stroke (HS) risk. While some variants increase susceptibility, others appear protective, leading to inconsistent findings. This meta-analysis systematically evaluates the APOE-HS association to clarify its role in stroke pathophysiology.

**Methods:**

A comprehensive literature search was conducted across multiple databases up to January 31, 2025, using the keywords: (“Apolipoprotein E” OR “APOE” OR “APOE genotype”) AND (“Single Nucleotide Polymorphisms” OR “SNP”) AND (“Hemorrhagic stroke” OR “HS” OR “Intracerebral Hemorrhage” OR “ICH”). The APOE ε3/ε3 genotype served as the reference genotype in all studies, and only those studies with ε3/ε3 genotype were included in the analysis. Pooled odds ratios (ORs) and 95% confidence intervals (CIs) were calculated, and statistical analyses were performed using STATA version 13.0 (StataCorp LLC, College Station, Texas, United States).

**Results:**

A total of 24 studies comprising 8,269 HS patients and 26,321 controls were included. Meta-analysis revealed a significant association of APOE ε2/ε2 (OR = 1.93, 95% CI = 1.32–2.81), ε4/ε4 (OR = 1.60, 95% CI = 1.21–2.13), ε2/ε4 (OR = 1.81, 95% CI = 1.34–2.44), ε2 (OR = 1.23, 95% CI = 1.12–1.35), and ε4 (OR = 1.31, 95% CI = 1.14–1.51) with an increased risk of HS.

**Conclusion:**

Our findings suggest that APOE ε2/ε2, ε2/ε4, ε2, and ε4/ε4 genotypes and the ε4 allele are associated with an elevated risk of HS. These results highlight the potential role of APOE genotypes in HS susceptibility and warrant further investigation.

## 1 Introduction

Stroke remains a leading global cause of mortality and disability, with approximately 15 million cases and 5 million deaths annually, placing a substantial burden on healthcare systems and caregivers (WHO, n.d.). Low-income countries like India face an escalating stroke crisis exacerbated by limited access to early intervention and preventive strategies (Feigin et al., 2021). Hemorrhagic stroke (HS), resulting from the rupture of intracranial blood vessels, is classified into intracerebral hemorrhage (ICH) and subarachnoid hemorrhage (SAH), contributing to 27.9 and 9.7% of new stroke cases, respectively (Unnithan et al., 2022; Feigin et al., 2021). Among these, ICH is notably more prevalent in Asian populations, with a disproportionately high mortality and disability rate. Despite its severe impact, the clinical understanding of HS remains incomplete, as multiple risk factors contribute to its onset and progression (Wang et al., 2022; de Rooij et al., 2007).

In recent years, genetic studies have played a crucial role in uncovering the underlying mechanisms of HS. Genome-wide association studies (GWAS) have identified potential candidate genes linked to HS susceptibility, shedding light on genetic influences in disease risk (Nie et al., 2019). Among these, the Apolipoprotein E (APOE) gene, located on chromosome 19, encodes a 317-amino acid glycoprotein involved in lipid metabolism and neuronal repair. The APOE gene exists in three major alleles—ε2, ε3, and ε4, forming six distinct genotypes (ε2/ε2, ε2/ε3, ε2/ε4, ε3/ε3, ε3/ε4, and ε4/ε4), with ε3/ε3 being the most common in the general population (Nie et al., 2019; Sudlow et al., 2006). APOE functions as a ligand for lipoprotein receptors, playing a critical role in plasma lipid regulation and transport (Sudlow et al., 2006; ScienceDirect Topics, n.d.). However, polymorphisms in APOE, particularly ε2 and ε4, have been associated with altered lipid metabolism and an increased risk of cerebrovascular diseases (Huang and Mahley, 2014).

Although prior studies have explored the relationship between APOE polymorphisms and stroke risk, findings remain inconsistent across populations and ethnicities (Huang and Mahley, 2014). Recent meta-analyses suggest that ε2 and ε4 alleles may be linked to an increased risk of ICH, but these associations vary across different demographic groups (Nie et al., 2019; Marini et al., 2019). Given these uncertainties, our study systematically analyzes and integrates existing evidence to provide a comprehensive assessment of the association between APOE genotypes and HS risk. By incorporating data from multiple studies, this meta-analysis aims to clarify genetic contributions to HS susceptibility and refine our understanding of APOE's role in stroke pathophysiology.

## 2 Methods

### 2.1 Literature search

We conducted this systematic review and meta-analysis in accordance with the Preferred Reporting Items for Systematic Reviews and Meta-Analyses (PRISMA) guidelines (Page et al., 2021). A comprehensive literature search was performed across multiple electronic databases, including PubMed, EMBASE, Cochrane Library, Scopus, and CINAHL, up to January 31, 2025. The search strategy incorporated relevant Medical Subject Headings (MeSH) and keywords, such as “Apolipoprotein E” (APOE), “APOE genotype,” “APOE ε2” and “APOE ε4,” alleles and their corresponding genotypes (ε2/ε2, ε2/ε3, ε2/ε4, ε3/ε4, and ε4/ε4), along with terms related to HS, ICH, cerebral hemorrhage, brain hemorrhage, and intracranial hemorrhage.

Two independent researchers (MN and AR) conducted the search, focusing exclusively on human studies without imposing language or publication date restrictions. To ensure a comprehensive dataset, we manually screened the reference lists of included studies, review articles, and previous meta-analyses for additional eligible studies.

#### 2.1.1 Study selection criteria

##### 2.1.1.1 Population (P)

Individuals aged ≥18 years with a clinically confirmed hemorrhagic stroke (HS) diagnosis based on CT or MRI.

##### 2.1.1.2 Intervention/exposure (I)

Studies reporting APOE allelic frequencies (ε2, ε3, and ε4) in both HS patients and control groups.

##### 2.1.1.3 Comparator (C)

Individuals without a history of stroke served as the control group.

##### 2.1.1.4 Outcomes (O)

- Primary outcome:
  ° Association between APOE genotypes (ε2/ε2 and ε4/ε4) and HS risk.

- Secondary outcomes:
  ° Association of ε2/ε3, ε2/ε4, and ε3/ε4 genotypes with HS risk.
  ° Association of ε2 and ε4 alleles with HS risk.
  ° HS risk based on APOE genotype across different population subgroups.
  ° HS risk based on hemorrhage location.

##### 2.1.1.5 Study selection (S)

- Inclusion criteria:
  ° Published case-control, cross-sectional, or cohort studies.
  ° A control group without prior stroke history must be included.

- Exclusion criteria:
  ° Duplicate publications or redundant data.

### 2.2 Data extraction

Based on the eligibility criteria, we screened the titles and abstracts of the literature from the available electronic databases. We gathered the following information from each included study: the first author's name, publication year, ethnicity, country, study design, study period, target population, mean age, sex, sample size (number of HS cases and healthy controls), type of hemorrhage, matching criteria, APOE genotyping method, allelic distribution of APOE genotypes in HS cases and healthy controls, and source of control. The ethnicities of the populations were classified as Asian or Caucasian. We requested any missing data from the corresponding authors of the articles. Any disagreements were resolved through consensus among all involved authors.

### 2.3 Data evaluation and validation

The genotyping studies included in the systematic review and meta-analysis were carefully reviewed for APOE genotypes and alleles to ensure the quality of their genotyping methodology and data capture. Most studies used the ε3 allele, and the ε3/ε3 genotype was used as the reference to assess associations between the ε2 and ε4 alleles and their genotypes and the ε3 allele. While reviewing the remaining studies that did not explicitly mention the use of a reference genotype, it was evident that the ε3 allele was used in the experiments, as the results all involved variations in the ε2 and ε4 alleles with risk association, and the ε3 allele and its genotype showed protective association or no association. Therefore, the authors of these studies were contacted to ascertain whether the ε3 allele and the ε3/ε3 genotype were used as genotyping references for the remaining alleles. The respective authors confirmed that the ε3 allele was used as a reference standard for all the analyses. Hence, our meta-analysis assessed the association of risk with the ε2 and ε4 alleles, and with the ε3 allele and the ε3/ε3 genotype as the reference.

### 2.4 Quality assessment

The methodological quality of the included studies was independently assessed by two reviewers (MN and AR) using the Newcastle-Ottawa Scale (NOS) (Kansagara et al., 2017), which evaluates selection, comparability, and outcome/exposure. Studies were categorized as high (≥7 points), moderate (4–6 points), or low ( ≤ 3 points) quality. Discrepancies were resolved through discussion with a third reviewer (PK).

### 2.5 Risk of bias assessment

Publication bias was assessed using Begg's funnel plot, while Egger's linear regression test evaluated plot asymmetry (Egger et al., 1997; Begg and Mazumdar, 1994). Sensitivity analyses were conducted to assess selection bias and heterogeneity by sequentially removing individual studies.

### 2.6 Genetic model of assessment

To assess the overall genetic risk association of the current APOE-based study with hemorrhagic stroke, both genotypic and allele-based models were used for comparative analyses against the reference allele ε3 and the genotype ε3/ε3, respectively. More importantly, the risk assessment in the genotypic model used a codominant comparison of APOE genotypes relative to the reference genotype. All the included studies were assessed for the Hardy-Weinberg equilibrium based on the availability of data from the control groups. The remaining studies were excluded from the final analysis.

### 2.7 Statistical analysis

A meta-analysis was performed to evaluate the association between APOE alleles/genotypes and HS risk, calculating pooled odds ratios (ORs) with 95% CIs. Heterogeneity was assessed using the *I*^2^ statistic, with < 50% indicating low and >50% suggesting moderate to high heterogeneity (Higgins et al., 2003). A fixed-effect model (Mantel and Haenszel, 1959) was applied for *I*^2^ < 50%, while a random-effect model (DerSimonian, 1996) was used for *I*^2^ > 50%. Subgroup analysis by ethnicity and meta-regression explored sources of heterogeneity. Statistical analyses were conducted in STATA 13.0, with *p* < 0.05 considered statistically significant. However, the results must be interpreted cautiously due to multiple testing, which can lead to a Type I error.

## 3 Results

### 3.1 Literature search

**Figure 1:**
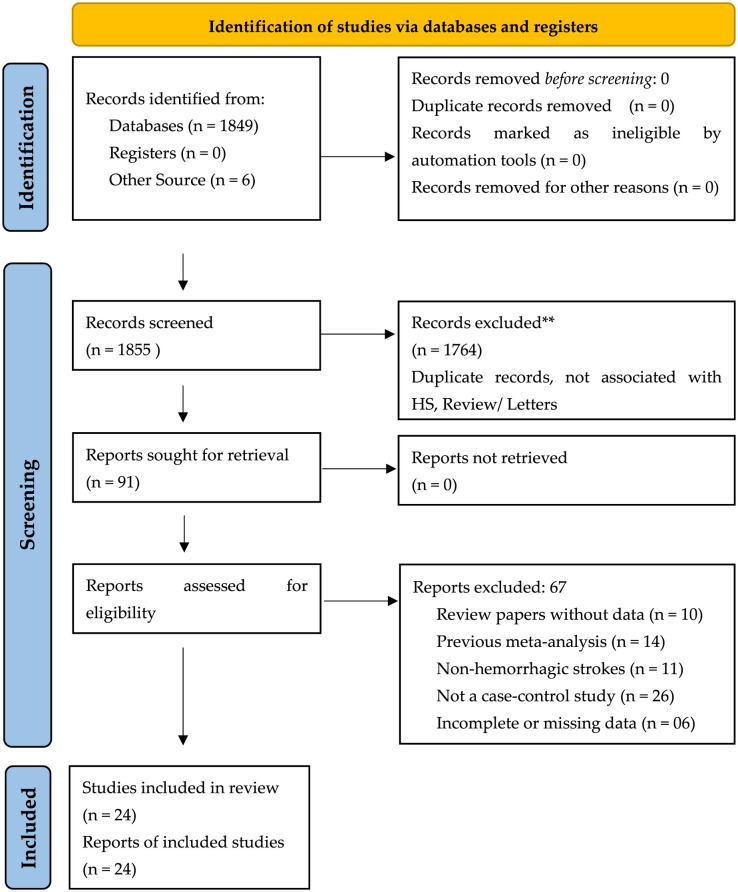
PRISMA flow diagram for the schematic representation of the included studies in our systematic review and meta-analysis. **Studies that were excluded based on screening based on relevance to the study objective, PICOT, and selection criteria.

The initial database search identified 1,849 articles, of which 1,764 were excluded after screening titles and abstracts. The full texts of 91 studies were assessed, with 67 excluded due to reviews, editorials, prior meta-analyses, non-hemorrhagic stroke studies, absence of control groups, or incomplete data. Finally, 24 studies met the eligibility criteria and were included in the systematic review and meta-analysis. The PRISMA flowchart (Figure 1) illustrates the study selection process.

### 3.2 Characteristics of eligible studies

The included studies, published between 1996 and 2024, examined multiple ethnicities across various geographic regions. Of the 24 studies, eight focused on Asian populations (Chowdhury et al., 2001; Ganaie et al., 2020; Das et al., 2016; Zhang et al., 2012; Kokubo et al., 2000; Misra et al., 2013; Nakata et al., 1997; Jiang et al., 2024), 19 on Caucasian (Catto et al., 1996; Duzenli et al., 2004; Martini et al., 2012; Seifert et al., 2006; Basun et al., 1996; Biffi et al., 2010; Garcia et al., 1999; Greenberg et al., 1996; McCarron and Nicoll, 1998; Tasdemir et al., 2008; Woo et al., 2013, 2005, 2002; Sawyer et al., 2018; Hostettler et al., 2022), three on African (Biffi et al., 2010; Sawyer et al., 2018; Atadzhanov et al., 2013), three on Hispanic (Biffi et al., 2010; Sawyer et al., 2018), and one on a mixed population (Sawyer et al., 2018). Among the Asian studies, three were conducted in India (Ganaie et al., 2020; Das et al., 2016; Misra et al., 2013), two in Japan and China each (Kokubo et al., 2000; Nakata et al., 1997), and one in Bangladesh (Chowdhury et al., 2001). Out of the 19 Caucasian studies, eight were performed in the American (USA) (Martini et al., 2012; Biffi et al., 2010; Greenberg et al., 1996; Woo et al., 2013, 2005, 2002; Sawyer et al., 2018) population, three in the British (Catto et al., 1996; McCarron and Nicoll, 1998; Hostettler et al., 2022) population, two in the Turkish (Duzenli et al., 2004; Tasdemir et al., 2008) population, two in the Swedish (Basun et al., 1996; Biffi et al., 2010) population, two in the Austrian (Seifert et al., 2006; Biffi et al., 2010) population, and one each in the Polish (Biffi et al., 2010) and Portuguese (Garcia et al., 1999) populations. Amongst the three African studies, two were conducted in the American (USA) (Biffi et al., 2010; Sawyer et al., 2018) population, and one was in the Zambian (Atadzhanov et al., 2013) population. Additionally, two of the three Hispanic studies were performed in the Spanish (Biffi et al., 2010) population, and one was in the American (USA) (Sawyer et al., 2018) population. Study quality varied, with six rated medium and 18 high. Seventeen were population-based, seven hospital-based, and most followed a case-control design, with two retrospective cohorts and one cross-sectional study. Table 1 provides an overview of baseline characteristics and quality assessments.

**Table 1:** Characteristics of the included studies in the meta-analysis depicting the association between *APOE* allele and genotype and the risk of hemorrhagic stroke in adult patients.

**S. no**.	**Study author and year**	**Country**	**Ethnicity**	**Study period**	**Study design**	**Type of hemorrhage**	**Sample size (case/control)**	**Genotyping method**	**Matching criteria**	**Source of control**	**NOS quality score**
1	Atadzhanov et al. (2013)	Zambia	African	June–December, 2010	CCS	HS	18/116	Taqman genotype sequencing	Age-sex	HB	8
2	Catto et al. (1996)	UK	Caucasian		CCS	PICH	60/289	PCR	Age-sex	PB	8
3	Chowdhury et al. (2001)	Bangladesh	Asian	April 1998–February 1999	CCS	HS	80/190	PCR-RFLP	Age-sex	PB	8
4	Das et al. (2016)	India	Asian	September 2007–December 2014	CCS	HS	250/620	PCR-RFLP	Age-sex	PB	9
5	Duzenli et al. (2004)	Turkey	Caucasian		CCS	HS	41/126	PCR	Age-sex	PB	6
6	Ganaie et al. (2020)	India	Asian	September 2012–2015	CCS	HS	68/108	PCR	Age-sex	HB	7
7	Martini et al. (2012)	USA	Caucasian	December 1997–August 2001; July 2002–December 2006	CCS	ICH	597/1,548	PCR	Age-sex	PB	7
8	Seifert et al. (2006)	Austria	Caucasian	October 1997–December 2000	CCS	Lobar and non-lobar ICH	193/280	PCR-RFLP		PB	4
9	Zhang et al. (2012)	China	Asian	2008–2010	CCS	ICH	180/180	PCR-RFLP		HB	8
10	Basun et al. (1996)	Sweden	Caucasian	1969–1994	RCS	Prior ICH	50/827	PCR		PB	6
						Subsequent ICH	87/827				
11	Biffi et al. (2010) GOCHA	USA	Caucasian		CCS	Lobar ICH	398/555	Genotype sequencing		PB	9
						Deep ICH	312/555				
	Biffi et al. (2010) GERFHS	USA	Caucasian	June 1997 and February 2000	CCS	Lobar ICH	203/1,304	Genotype sequencing		PB	
						Deep ICH	337/1,304				
	Biffi et al. (2010) JUHSS	Poland	Caucasian	April 1998–May 2007	CCS	Lobar ICH	102/429	Genotype sequencing		PB	
						Deep ICH	130/429				
	Biffi et al. (2010) MUG-ICH	Austria	Caucasian	2002 and 2006	CCS	Lobar ICH	77/1,023	Genotype sequencing		PB	
						Deep ICH	114/1,023				
	Biffi et al. (2010) HM-ICH	Spain	Hispanic	January to December 1996	CCS	Lobar ICH	66/185	Genotype sequencing		PB	
						Deep ICH	103/185				
	Biffi et al. (2010) LUHSS	Sweden	Caucasian	October 1997 and December 2000	CCS	Lobar ICH	42/161	Genotype sequencing		PB	
						Deep ICH	89/161				
	Biffi et al. (2010) VHH-ICH	Spain	Hispanic	10-year period	CCS	Lobar ICH	43/87	Genotype sequencing		PB	
						Deep ICH	-				
	Biffi et al. (2010) US-AA	USA	African		CCS	Lobar ICH	63/297	Genotype sequencing		PB	
						Deep ICH	110/297				
12	Garcia et al. (1999)	Portugal	Caucasian		CCS	Lobar ICH	24/24	PCR	Age-sex	PB	9
						Deep ICH	24/24		Age-sex		
13	Greenberg et al. (1996)	USA	Caucasian		CCS	Lobar Hemorrhage	45/3,798	PCR		PB	5
						Non-lobar Hemorrhage	18/3,798				
14	Kokubo et al. (2000)	Japan	Asian	November 1997–June 1999	CCS	ICH	84/1,126	PCR		PB	6
15	McCarron and Nicoll (1998)	UK	Caucasian		CCS	SAH	96/406	PCR		HB	5
						Deep Hemorrhage	71/406				
						CAA Hemorrhage	40/406				
16	Misra et al. (2013)	India	Asian		CCS	RICH	33/188	PCR	Age-sex	PB	8
						NRICH	101/188		Age-sex		
17	Nakata et al. (1997)	Japan	Asian	July 1992–December 1995	CCS	HS	38/38	PCR	Age-sex	HB	8
18	Sawyer et al. (2018)	USA	Mixed	September 2009–July 2016	CCS	Total ICH	907/2,660	PCR, Taqman SNP genotyping	Age-sex	PB	9
			Caucasian			White ICH	401/979		Age-sex		
			Hispanic			Hispanic ICH	269/795		Age-sex		
			African			Black ICH	237/886		Age-sex		
19	Tasdemir et al. (2008)	Turkey	Caucasian	Septeber 2005–June 2006	CCS	PICH	35/30	PCR, ApoE detection kit	Age-sex	HB	9
20	Woo et al. (2005)	USA	Caucasian	May 1997–December 2002	CCS	Lobar ICH	102/187	Taqman assay	Age-sex	PB	7
21	Woo et al. (2013)	USA	Caucasian	July 2008–June 2013	CCS	Lobar ICH	204/508	PCR	Age-sex	PB	9
						Non-lobar ICH	354/936		Age-sex		
22	Woo et al. (2002)	USA	Caucasian	June 1997 and February 1998	CCS	All ICH	188/366	PCR	Age-sex	PB	9
				June 1997 and February 1999		Lobar ICH	67/131		Age-sex		
				June 1997 and February 2000		Non-lobar ICH	121/235		Age-sex		
23	Hostettler et al. (2022)	UK	Caucasian		CCS	ICH	907/2,636	Genotype sequencing		PB	7
24	Jiang et al. (2024)	China	Asian	September 2020–September 2022	RCS	ICH	153/60	QF-PCR		HB	7

RCS, Retrospective cohort study; CCS, case-control study; HB, hospital based; PB, population based; HS, hemorrhagic stroke; ICH, intracerebral hemorrhage; PCR, polymerase chain reaction; RFLP, restriction fragment length polymorphism; UK, United Kingdom; USA, United States of America; SNP, single nucleotide polymorphism; PICH, primary intracerebral hemorrhage; SAH, subarachnoid hemorrhage; CAA, cerebral amyloid angiopathy; RICH, recurrent intracerebral hemorrhage; NRICH, non-recurrent intracerebral hemorrhage; APOE, apolipoprotein E; QF-PCR, quantitative fluorescent polymerase chain reaction.

### 3.3 Primary outcomes

#### 3.3.1 Association between the APOE ε2/ε2 genotype and the risk of hemorrhagic stroke

**Figure 2:**
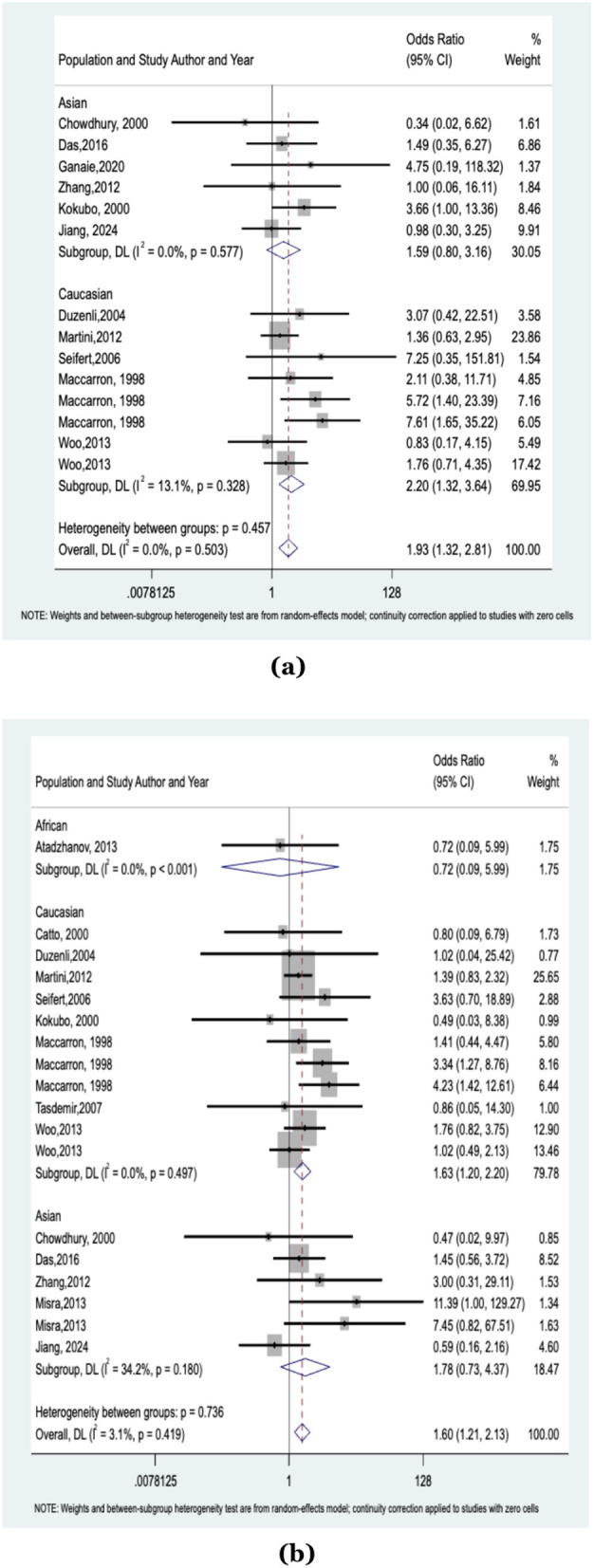
**(a)** Forest plot showing the association between the ε2/ε2 genotype of the *APOE* gene and the risk of hemorrhagic stroke; **(b)** Forest plot showing the association between the ε4/ε4 genotype of the *APOE* gene and the risk of hemorrhagic stroke.

The meta-analysis included 11 studies with 2,411 cases and 6,900 controls assessing the association between the ε2/ε2 genotype of the APOE gene and hemorrhagic stroke (HS) risk. A significant overall association was observed (OR: 1.93, 95% CI = 1.32–2.81, *I*^2^ = 0%). Subgroup analysis by ethnicity revealed no significant association in the Asian population (815 cases, 2,284 controls; OR: 1.59, 95% CI = 0.80–3.16, *I*^2^ = 0%), while the Caucasian population (1,596 cases, 4,616 controls) showed a significant association (OR: 2.20, 95% CI = 1.32–3.64, *I*^2^ = 13.1%). The results are illustrated in Figure 2a and Table 2.

**Table 2:** Effect size of the overall population and subgroups within the included studies of our meta-analysis.

**Population**	**Sample size (cases/control)**	**OR (95% CI)**	***I*^2^ (%)**	**Publication bias (Egger *p*-value)**	**Meta-regression (*p*-value)**
Overall (ε2/ε2)	2,411/6,900	**1.93 (1.32, 2.81)**	0	0.494	0.845
Asian (ε2/ε2)	815/2,284	1.59 (0.80, 3.16)	0		
Caucasian (ε2/ε2)	1,596/4,616	**2.20 (1.32, 3.64)**	13.1		
Overall (ε4/ε4)	2,590/7,603	**1.60 (1.21, 2.13)**	3.1	0.760	0.142
Asian (ε4/ε4)	797/1,426	1.78 (0.73, 4.37)	34.2		
Caucasian (ε4/ε4)	1,775/6,061	**1.63 (1.20, 2.20)**	0		
Overall (ε2/ε3)	2,572/7,383	0.95 (0.54, 1.65)	90.7	0.599	0.225
Asian (ε2/ε3)	815/2,284	0.52 (0.15, 1.83)	91.8		
Caucasian (ε2/ε3)	1,739/4,983	1.43 (0.79, 2.59)	88.9		
Overall (ε2/ε4)	3,652/10,525	**1.81 (1.34, 2.44)**	48.3	0.097	0.827
Asian (ε2/ε4)	848/2,472	**3.70 (1.25, 10.97)**	69.5		
Caucasian (ε2/ε4)	2,786/7,937	**1.44 (1.19, 1.75)**	0		
Overall (ε3/ε4)	2,706/7,759	1.04 (0.86, 1.26)	52.8	0.371	0.758
Asian (ε3/ε4)	949/2,660	1.22 (0.67, 2.23)	77.3		
Caucasian (ε3/ε4)	1,739/4,983	1.02 (0.88, 1.17)	7.1		
Overall (ε2)	5,287/24,288	**1.23 (1.12, 1.35)**	0	0.229	0.048
Asian (ε2)	750/1,512	1.15 (0.85–1.56)	0		
Caucasian (ε2)	3,906/20,872	**1.30 (1.17, 1.44)**	0		
African (ε2)	255/1,002	1.01 (0.72, 1.42)	0		
Overall ICH (ε2)	5,287/24,288	**1.23 (1.12, 1.35)**	0	0.229	0.048
Hemorrhagic stroke (ε2)	1,666/5,164	**1.20 (1.03, 1.41)**	0		
Lobar ICH (ε2)	1,192/4,303	**1.36 (1.11, 1.68)**	0		
Deep ICH (ε2)	1,255/7,776	1.04 (0.83, 1.31)	0		
Overall (ε4)	5,435/24,428	**1.31 (1.14, 1.51)**	59.0	0.8	0.349
Asian (ε4)	903/1,572	1.80 (0.82, 3.91)	86.1		
Caucasian (ε4)	3,906/20,872	**1.21 (1.09, 1.35)**	13.1		
African (ε4)	255/1,002	1.04 (0.80, 1.34)	0		
Overall ICH (ε4)	2,575/16,192	**1.31 (1.14, 1.51)**	59.0	0.8	0.349
Hemorrhagic stroke (ε4)	415/1,008	1.26 (0.97, 1.64)	0		
ICH (ε4)	1,324/4,002	1.27 (0.80, 2.02)	78.8		
Lobar ICH (ε4)	1,187/4,383	**1.49 (1.26, 1.78)**	0		
Deep ICH (ε4)	1,255/7,776	1.13 (0.94, 1.36)	0		

The included studies for each analysis, along with their related details for outcome assessment, are provided in the Supplementary Table 2.

Bold values indicate statistically significant values at 95%CI (*p*-value < 0.05).

#### 3.3.2 Association between the APOE ε4/ε4 genotype and the risk of hemorrhagic stroke

For the ε4/ε4 genotype, 14 studies with 2,590 cases and 7,603 controls were analyzed. A significant association was found between the ε4/ε4 genotype and increased HS risk (OR: 1.60, 95% CI = 1.21–2.13, *I*^2^ = 3.1%). Subgroup analysis showed a strong association in the Caucasian population (OR: 1.63, 95% CI = 1.20–2.20, *I*^2^ = 0%), while no significant association was observed in the Asian subgroup (OR: 1.78, 95% CI = 0.73–4.37, *I*^2^ = 34.2%). The single African study was excluded from the subgroup analysis. All findings are depicted in Figure 2b and Table 2.

### 3.4 Secondary outcomes

#### 3.4.1 Association between APOE ε2/ε3 genotype and the risk of hemorrhagic stroke

In this meta-analysis, 15 studies involving 2,572 cases and 7,383 control subjects were analyzed to evaluate the ε2/ε3 genotype of the *APOE* gene. No significant association was found between the presence of the ε2/ε3 genotype and the risk of HS for the overall population (OR: 0.95, 95% CI = 0.54–1.65, *I*^2^ = 90.7%). During the subgroup analysis based on the ethnicities of the subjects, no significant association was observed in the Asian (OR: 0.52, 95% CI = 0.15–1.83, *I*^2^ = 91.8%) and Caucasian (OR: 1.43, 95% CI = 0.79–2.59, *I*^2^ = 88.9%) populations regarding the ε2/ε3 genotype and HS risk. The results are presented in Supplementary Figure S2-1 and Table 2.

#### 3.4.2 Association between APOE ε2/ε4 genotype and the risk of hemorrhagic stroke

A total of 17 studies involving 3,652 cases and 10,525 control subjects were included in the meta-analysis to understand the impact of the ε2/ε4 genotype of the *APOE* gene on HS. We observed a significant association between the occurrence of the ε2/ε4 genotype of the *APOE* gene and the risk of HS in the overall population (OR: 1.81, 95% CI = 1.34–2.44, *I*^2^ = 48.3%). Additionally, during the subgroup analysis based on the ethnicities of the individual studies, both the Asian (OR: 3.70, 95% CI = 1.25–10.97, *I*^2^ = 69.5%) and Caucasian (OR: 1.44, 95% CI = 1.19–1.75, *I*^2^ = 0%) subgroups also demonstrated an association between the ε2/ε4 genotype and HS risk. The results are presented in Supplementary Figure S2-2 and Table 2.

#### 3.4.3 Association between APOE ε3/ε4 genotype and the risk of hemorrhagic stroke

To analyze the impact of the ε3/ε4 genotype of the APOE gene on HS, we included 16 studies encompassing 2,706 cases and 7,759 control subjects. There was no significant association between HS risk and the presence of the ε3/ε4 genotype of the APOE gene in the overall population (OR: 1.04, 95% CI = 0.86–1.26, *I*^2^ = 52.8%). Subgroup analyses of ethnic populations across the different studies also demonstrated no significant association between the ε3/ε4 genotype and HS risk among the Asian (OR: 1.22, 95% CI = 0.67–2.23, *I*^2^ = 77.3%) and Caucasian (OR: 1.02, 95% CI = 0.88–1.17, *I*^2^ = 7.1%) populations. The results are illustrated in Supplementary Figure S2-3 and Table 2.

#### 3.4.4 Association between the APOE ε2 allele and the risk of hemorrhagic stroke

To understand the impact of the ε2 allele of the APOE gene on hemorrhagic stroke (HS), we included 16 studies comprising 5,287 cases and 24,288 control subjects in this meta-analysis. We observed a significant association between the ε2 allele of the APOE gene and the risk of hemorrhagic stroke in the overall population (OR: 1.23, 95% CI = 1.12–1.35, *I*^2^ = 0%). A subgroup analysis revealed a significant association between the ε2 allele and HS risk in the Caucasian population (OR: 1.30, 95% CI = 1.17–1.44, *I*^2^ = 0%). However, no significant relationship was found in the Asian (OR: 1.15, 95% CI = 0.85–1.56, *I*^2^ = 0%) and African (OR: 1.01, 95% CI = 0.72–1.42, *I*^2^ = 0%) subgroups regarding the association between the ε2 allele and the risk of HS. The mixed and Hispanic populations could not be subgrouped because only one study was available. The results are shown in Supplementary Figure S2-4 and Table 2.

#### 3.4.5 Association between the APOE ε4 allele and the risk of hemorrhagic stroke

This meta-analysis included 17 studies with 5,435 cases and 24,428 controls to assess the impact of the ε4 allele on hemorrhagic stroke (HS) risk. A significant association was found in the overall population (OR: 1.31, 95% CI = 1.14–1.51, *I*^2^ = 59.0%). Subgroup analysis revealed a significant association in the Caucasian population (OR: 1.21, 95% CI = 1.09–1.35, *I*^2^ = 13.1%), while no significant association was observed in Asian (OR: 1.80, 95% CI = 0.82–3.91, *I*^2^ = 86.1%) or African (OR: 1.04, 95% CI = 0.80–1.34, *I*^2^ = 0%) populations. Due to limited data, subgroup analysis for Hispanic and mixed populations was not feasible. The results are illustrated in Supplementary Figure S2-6 and Table 2.

### 3.5 Subgroup analysis by hemorrhage location

The APOE ε2 allele was assessed based on the location of the hemorrhagic stroke, encompassing 5,287 cases and 24,288 control subjects from 16 included studies. The locations were categorized into lobar, deep, mixed, non-lobar intracerebral hemorrhage (ICH), and rapidly involuting (RICH) or non-involuting (NICH) congenital hemangiomas. Additionally, those who could not be classified based on location were grouped under the hemorrhagic stroke category. It was found that the ε2 allele of the APOE gene had a significant association with the risk of hemorrhagic stroke for the overall location of all intracerebral hemorrhages (OR: 1.23, 95% CI = 1.12–1.35, *I*^2^ = 0%). Moreover, there was a significant association of the ε2 allele with hemorrhagic stroke risk for lobar ICH (OR: 1.36, 95% CI = 1.11–1.68, *I*^2^ = 0%). However, no significant association was observed for the deep lobar location of the hemorrhage concerning the ε2 allele and hemorrhagic stroke risk (OR: 1.04, 95% CI = 0.83–1.31, *I*^2^ = 0%). The mixed, non-lobar ICH, RICH, and NICH studies were not categorized separately because they consisted of only one study. Those with undefined locations of hemorrhagic stroke exhibited a significant association between the risk of intracerebral hemorrhage and the ε2 allele of APOE (OR: 1.20, 95% CI = 1.03–1.41, *I*^2^ = 0%). The overall results are illustrated in Supplementary Figure S2-5 and Table 2.

The association of the ε4 allele with HS risk across different hemorrhage locations was also analyzed in 17 studies with the same dataset. A significant association was found across all locations (OR: 1.31, 95% CI = 1.14–1.51, *I*^2^ = 59.0%), with a stronger link for lobar ICH (OR: 1.49, 95% CI = 1.26–1.78, *I*^2^ = 0%). However, no significant association was observed for deep ICH (OR: 1.13, 95% CI = 0.94–1.36, *I*^2^ = 0%), undefined hemorrhagic stroke (OR: 1.26, 95% CI = 0.97–1.64, *I*^2^ = 0%), or ICH with unspecified location (OR: 1.27, 95% CI = 0.80–2.02, *I*^2^ = 78.8%). Subgroup analysis for mixed, non-lobar ICH, RICH, and NICH was not possible due to limited studies. Results are depicted in Supplementary Figure S2-7 and Table 2.

### 3.6 Publication bias

Begg's funnel plot and Egger's test were used to assess publication bias. The symmetrical funnel plot for ε2/ε2 and ε4/ε4 genotypes suggested no bias (Figures 3a, b). Egger's test confirmed this (*p* = 0.494 for ε2/ε2 and 0.760 for ε4/ε4). Secondary outcomes, including ε2/ε3, ε2/ε4, ε3/ε4 genotypes and ε2, ε4 alleles, also showed no publication bias (Supplementary Figures S2-8–12). Egger's *p*-values for all analyses are summarized in Table 2.

**Figure 3:**
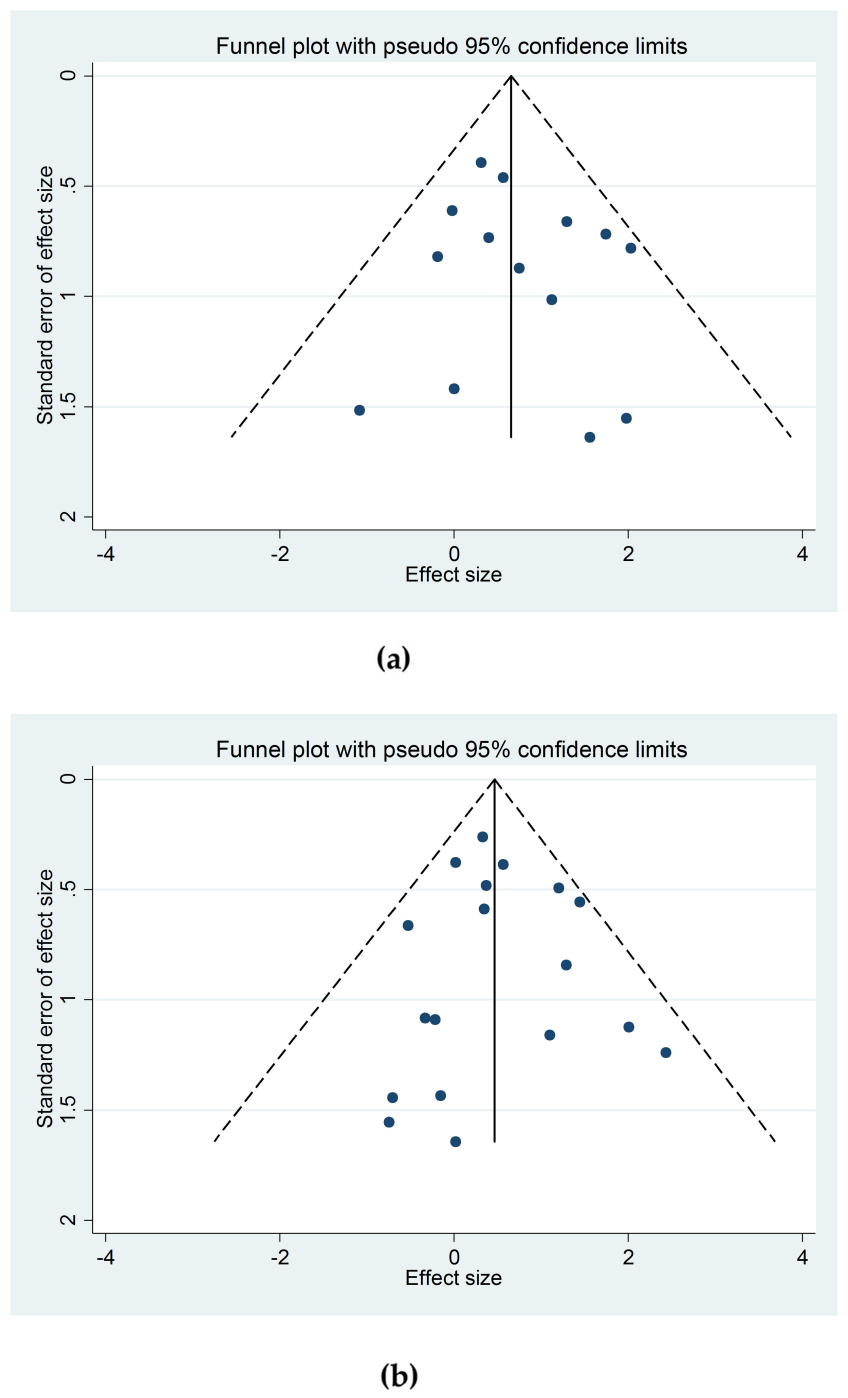
**(a)** Begg's Funnel plot for assessing the publication bias of the studies depicting the association between the ε2/ε2 genotype of the *APOE* gene and the risk of hemorrhagic stroke; **(b)** Begg's Funnel plot for assessing the publication bias of the studies depicting the association between the ε4/ε4 genotype of the *APOE* gene and the risk of hemorrhagic stroke.

### 3.7 Meta-regression analysis

Meta-regression analysis was performed to assess the effect size based on study quality. No significant deviations were observed in the association between the ε2/ε2 and ε4/ε4 genotypes of the APOE gene and HS risk (Figures 4a, b). Similarly, analyses for ε2/ε3, ε2/ε4, ε3/ε4 genotypes and ε4 allele showed no notable deviations. However, the ε2 allele exhibited some deviation (*p* = 0.048), suggesting cautious interpretation of its association with HS risk. Table 2 summarizes the meta-regression *p*-values, with additional details in Supplementary Figures S2-13–17.

**Figure 4:**
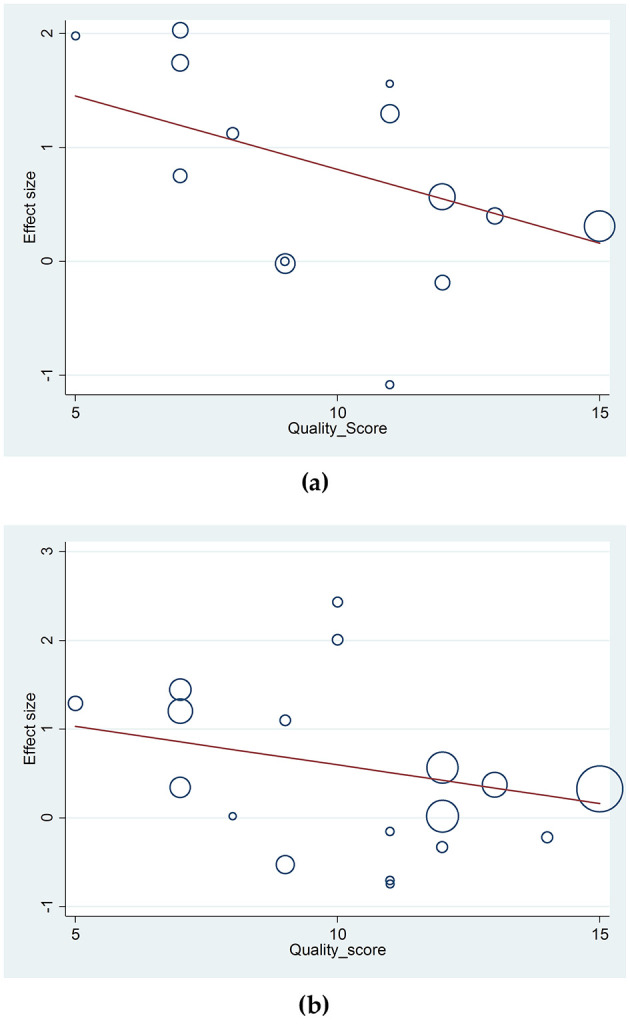
**(a)** Meta-regression analysis of the included studies to assess the effect of the association between the ε2/ε2 genotype of the *APOE* gene and the risk of hemorrhagic stroke; **(b)** Meta-regression analysis of the included studies to assess the effect of the association between the ε4/ε4 genotype of the *APOE* gene and the risk of hemorrhagic stroke.

### 3.8 Sensitivity analysis

Sensitivity analysis using the Leave-one-out method showed no significant changes in effect size for the ε2/ε2 and ε4/ε4 genotypes of the APOE gene and HS risk (Figures 5a, b). Similarly, no notable variations were observed for ε2/ε3, ε2/ε4, ε3/ε4 genotypes or ε2 and ε4 alleles (Supplementary Figures S2-18–22), confirming the robustness of the findings.

**Figure 5:**
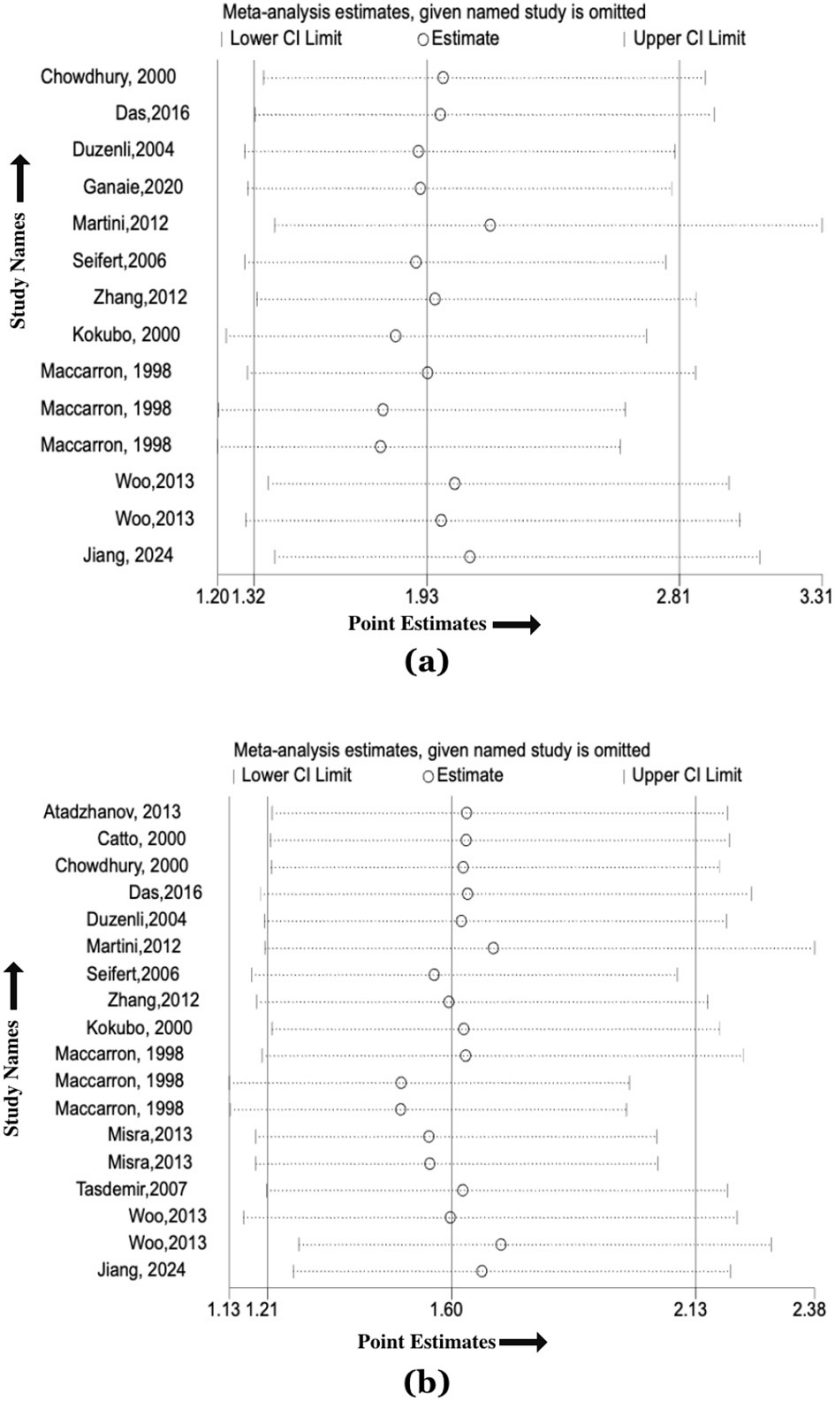
**(a)** Sensitivity analysis plot evaluating the effect of the association between the ε2/ε2 genotype of the *APOE* gene and the association of hemorrhagic stroke risk; **(b)** Sensitivity analysis plot evaluating the effect of the association between the ε4/ε4 genotype of the *APOE* gene and the association of hemorrhagic stroke risk.

## 4 Discussion

This systematic review and meta-analysis included 24 studies with 8,269 HS cases and 26,321 controls across various ethnicities, primarily Caucasian and Asian. Significant associations were observed between ε2/ε2, ε4/ε4, and ε2/ε4 genotypes and HS risk. Both ε2 and ε4 alleles were linked to increased HS risk, with no significant associations for other APOE variants.

### 4.1 Ethnicity-based and hemorrhage location subgroup analyses

Subgroup analysis confirmed significant associations between ε2/ε2, ε4/ε4, and ε2/ε4 genotypes and HS risk in Caucasian populations. In Asian populations, only the ε2/ε4 genotype showed a significant risk association. Further analysis by hemorrhage location revealed that ε2 and ε4 alleles were strongly linked to lobar hemorrhages, whereas no association was found with deep hemorrhages.

### 4.2 Comparison with previous meta-analyses

Our findings align with (Nie et al. 2019), which identified ε2 and ε4 alleles as risk factors for intracerebral hemorrhage (ICH) in Caucasians, but with a significantly larger sample (24 vs. eight studies). Similarly, our results corroborate (Marini et al. 2019), who found a strong link between ε2 and ε4 alleles and lobar ICH in Caucasians, with our larger dataset reinforcing their conclusions. Additionally, (Pan et al. 2021) demonstrated poor functional outcomes in ICH patients with the ε4 allele, further supporting our findings.

### 4.3 APOE and its broader clinical implications

Beyond HS, the APOE gene has been extensively studied in neurodegenerative diseases, including Alzheimer's disease, where the ε4 allele is a well-established risk factor across ethnicities, including Indian populations (Farrer et al., 1997; Agarwal and Tripathi, 2014). APOE polymorphisms are also linked to lipid metabolism, cardiovascular diseases, and stroke risk, with associations found between APOE alleles and cholesterol levels, atherosclerosis, and oxidative injury protection (Ozen et al., 2022).

### 4.4 Unresolved mechanisms and future directions

Conflicting evidence exists regarding the mechanisms behind APOE polymorphisms and HS risk. While ε2 and ε4 alleles are implicated in abnormal lipid metabolism, blood-brain barrier disruption, and vasogenic edema, some studies suggest APOE presence may protect against oxidative neuronal damage. Our study, the most extensive meta-analysis to date on APOE polymorphisms and HS, strengthens the link between ε2 and ε4 alleles and HS risk (Biffi et al., 2010; Zhang et al., 2014; Sudlow et al., 2006). These findings provide a robust foundation for future observational studies on APOE genotypes and their role in HS, offering potential clinical implications for risk stratification and targeted interventions. These findings can provide a guideline for conducting future observational studies on *APOE* genotypes to ascertain the relationship with the risk of HS, thereby facilitating clinical transition.

### 4.5 Limitations

Despite its comprehensive approach, this study has some limitations. Key comorbidities like hypertension, diabetes, and prior stroke or cardiac disease were not accounted for, potentially influencing outcomes. False discovery rates were not assessed, which may affect comparisons. Some heterogeneity was noted, with meta-regression indicating bias; however, this was addressed through the random-effects model and sensitivity analyses. Importantly, the study demonstrated minimal heterogeneity and no publication bias, reinforcing the reliability of the pooled effect sizes. The lack of risk factor, confounder, and covariate data in the included studies limits the generalizability of the study outcomes and, hence, the results must be interpreted with caution.

## 5 Conclusions

This meta-analysis provides a comprehensive evaluation of the association between APOE alleles and hemorrhagic stroke (HS) risk. Significant associations were identified for ε2/ε2, ε4/ε4, and ε2/ε4 genotypes, as well as ε2 and ε4 alleles, with HS risk in both the overall and Caucasian populations. Additionally, a significant link between the ε2/ε4 genotype and HS risk was observed in the Asian population. These findings highlight the need for large-scale prospective studies to further validate these associations.

## Data Availability

The original contributions presented in the study are included in the article/Supplementary material, further inquiries can be directed to the corresponding author.
