# Supplementary material for: The APOE paradox: divergent genetic influences on hemorrhagic stroke risk—A meta-analysis
Source: Front Stroke. 2026 Mar 4;5:1684121. doi: 10.3389/fstro.2026.1684121 (PMC12995800; doi:10.3389/fstro.2026.1684121)
Supplement: Supplementary file 2 [file Supplementary_file_2.docx]

**Figure S2-1:** Forest plot showing the association between the ε2/ε3 genotype of the *APOE* gene and the risk of hemorrhagic stroke.

**Figure S2-2:** Forest plot showing the association between the ε2/ε4 genotype of the *APOE* gene and the risk of hemorrhagic stroke.

**Figure S2-3:** Forest plot showing the association between the ε3/ε4 genotype of the *APOE* gene and the risk of hemorrhagic stroke.

**Figure S2-4:** Forest plot showing the association between the ε2 allele of the *APOE* gene and the risk of hemorrhagic stroke.

**Figure S2-5:** Forest plot showing the association between the ε2 allele of the *APOE* gene and the risk of hemorrhagic stroke based on the location of the hemorrhage.

**Figure S2-6:** Forest plot showing the association between the ε4 allele of the *APOE* gene and the risk of hemorrhagic stroke.

**Figure S2-7:** Forest plot showing the association between the ε4 allele of the *APOE* gene and the risk of hemorrhagic stroke based on the location of the hemorrhage.

**Figure S2-8:** Begg’s Funnel plot for assessing the publication bias of the studies depicting the association between the ε2/ε3 genotype of the *APOE* gene and the risk of hemorrhagic stroke.

**Figure S2-9:** Begg’s Funnel plot for assessing the publication bias of the studies depicting the association between the ε2/ε4 genotype of the *APOE* gene and the risk of hemorrhagic stroke.

**Figure S2-10:** Begg’s Funnel plot for assessing the publication bias of the studies depicting the association between the ε3/ε4 genotype of the *APOE* gene and the risk of hemorrhagic stroke.

**Figure S2-11:** Begg’s Funnel plot for assessing the publication bias of the studies depicting the association between the ε2 allele of the *APOE* gene and the risk of hemorrhagic stroke.

**Figure S2-12:** Begg’s Funnel plot for assessing the publication bias of the studies depicting the association between the ε4 allele of the *APOE* gene and the risk of hemorrhagic stroke.

**Figure S2-13:** Meta-regression analysis of the included studies to assess the effect of the association between the ε2/ε3 genotype of the *APOE* gene and the risk of hemorrhagic stroke.

**Figure S2-14:** Meta-regression analysis of the included studies to assess the effect of the association between the ε2/ε4 genotype of the *APOE* gene and the risk of hemorrhagic stroke.

**Figure S2-15:** Meta-regression analysis of the included studies to assess the effect of the association between the ε3/ε4 genotype of the *APOE* gene and the risk of hemorrhagic stroke.

**Figure S2-16:** Meta-regression analysis of the included studies to assess the effect of the association between the ε2 allele of the *APOE* gene and the risk of hemorrhagic stroke.

**Figure S2-17:** Meta-regression analysis of the included studies to assess the effect of the association between the ε4 allele of the *APOE* gene and the risk of hemorrhagic stroke.

**Figure S2-18:** Sensitivity analysis plot evaluating the effect of the association between the ε2/ε3 genotype of the *APOE* gene and the association of hemorrhagic stroke risk.

**Figure S2-19:** Sensitivity analysis plot evaluating the effect of the association between the ε2/ε4 genotype of the *APOE* gene and the association of hemorrhagic stroke risk.

**Figure S2-20:** Sensitivity analysis plot evaluating the effect of the association between the ε3/ε4 genotype of the *APOE* gene and the association of hemorrhagic stroke risk.

**Figure S2-21:** Sensitivity analysis plot evaluating the effect of the association between the ε2 allele of the *APOE* gene and the association of hemorrhagic stroke risk.

**Figure S2-22:** Sensitivity analysis plot evaluating the effect of the association between the ε4 allele of the *APOE* gene and the association of hemorrhagic stroke risk.
