# Supplementary material for: The APOE paradox: divergent genetic influences on hemorrhagic stroke risk—A meta-analysis
Source: Front Stroke. 2026 Mar 4;5:1684121. doi: 10.3389/fstro.2026.1684121 (PMC12995800; doi:10.3389/fstro.2026.1684121)
Supplement: Supplementary file 3 [file Table_1.docx]

**Table 1: Association between the APOE ε2/ε2 genotype and the risk of hemorrhagic stroke**

| **Study Author and Year** | **Ethnicity** | **Population** | **Type of Hemorrhage** | **Cases** | **Controls** | **APOE2.2_Cases** | **APOE2.2_Controls** |
| --- | --- | --- | --- | --- | --- | --- | --- |
| Chowdhury, 2000 | 1 | Asian | Cerebral Hemorrhage | 80 | 190 | 0 | 3 |
| Das,2016 | 1 | Asian | Hemorrhagic stroke | 250 | 620 | 3 | 5 |
| Duzenli,2004 | 2 | Caucasian | Hemorrhagic stroke | 41 | 126 | 2 | 2 |
| Ganaie,2020 | 1 | Asian | Hemorrhagic stroke | 68 | 108 | 1 | 0 |
| Martini,2012 | 2 | Caucasian | Intracerebral Hemorrhage | 597 | 1548 | 10 | 19 |
| Seifert,2006 | 2 | Caucasian | Lobar and Non lobar ICH | 193 | 280 | 2 | 0 |
| Zhang,2012 | 1 | Asian | Intracerebral Hemorrhage | 180 | 180 | 1 | 1 |
| Kokubo, 2000 | 1 | Asian | ICH | 84 | 1126 | 3 | 11 |
| Maccarron, 1998 | 2 | Caucasian | SAH | 96 | 406 | 2 | 4 |
| Maccarron, 1998 | 2 | Caucasian | Deep Hemorrhage | 71 | 406 | 4 | 4 |
| Maccarron, 1998 | 2 | Caucasian | CAA Hemorrhage | 40 | 406 | 3 | 4 |
| Woo,2013 | 2 | Caucasian | Lobar ICH | 204 | 508 | 2 | 6 |
| Woo,2013 | 2 | Caucasian | Non Lobar ICH | 354 | 936 | 8 | 12 |
| Jiang, 2024 | 1 | Asian | ICH | 153 | 60 | 10 | 4 |

**Table 2: Association between the APOE ε4/ε4 genotype and the risk of hemorrhagic stroke**

| **Study Author and Year** | **Ethnicity** | **Population** | **Type of Hemorrhage** | **Cases** | **Controls** | **APOE4.4_Cases** | **APOE4.4_Controls** |
| --- | --- | --- | --- | --- | --- | --- | --- |
| Atadzhanov, 2013 | 3 | African | Hemorrhagic stroke | 18 | 116 | 1 | 9 |
| Catto, 2000 | 2 | Caucasian | PICH | 60 | 289 | 1 | 6 |
| Chowdhury, 2000 | 1 | Asian | Cerebral Hemorrhage | 80 | 190 | 0 | 2 |
| Das,2016 | 1 | Asian | Hemorrhagic stroke | 250 | 620 | 7 | 12 |
| Duzenli,2004 | 2 | Caucasian | Hemorrhagic stroke | 41 | 126 | 0 | 1 |
| Martini,2012 | 2 | Caucasian | Intracerebral Hemorrhage | 597 | 1548 | 23 | 43 |
| Seifert,2006 | 1 | Caucasian | Lobar and Non lobar ICH | 193 | 280 | 5 | 2 |
| Zhang,2012 | 1 | Asian | Intracerebral Hemorrhage | 180 | 180 | 3 | 1 |
| Kokubo, 2000 | 2 | Caucasian | ICH | 84 | 1126 | 0 | 13 |
| Maccarron, 1998 | 2 | Caucasian | SAH | 96 | 406 | 4 | 12 |
| Maccarron, 1998 | 2 | Caucasian | Deep Hemorrhage | 71 | 406 | 7 | 12 |
| Maccarron, 1998 | 2 | Caucasian | CAA Hemorrhage | 40 | 406 | 5 | 12 |
| Misra,2013 | 1 | Asian | RICH | 33 | 188 | 2 | 1 |
| Misra,2013 | 1 | Asian | NRICH | 101 | 188 | 4 | 1 |
| Tasdemir,2007 | 2 | Caucasian | PICH | 35 | 30 | 1 | 1 |
| Woo,2013 | 2 | Caucasian | Lobar ICH | 204 | 508 | 12 | 17 |
| Woo,2013 | 2 | Caucasian | Non Lobar ICH | 354 | 936 | 10 | 26 |
| Jiang, 2024 | 1 | Asian | ICH | 153 | 60 | 6 | 4 |

**Table 3: Association between APOE ε2/ε3 genotype and the risk of hemorrhagic stroke**

| **Study Author and Year** | **Ethnicity** | **Population** | **Type of Hemorrhage** | **Cases** | **Controls** | **APOE2.3_Cases** | **APOE2.3_Controls** |
| --- | --- | --- | --- | --- | --- | --- | --- |
| Atadzhanov, 2013 | 3 | African | Hemorrhagic stroke | 18 | 116 | 0 | 25 |
| Catto, 2000 | 2 | Caucasian | PICH | 60 | 289 | 3 | 37 |
| Chowdhury, 2000 | 1 | Asian | Cerebral Hemorrhage | 80 | 190 | 2 | 6 |
| Das,2016 | 1 | Asian | Hemorrhagic stroke | 250 | 620 | 7 | 560 |
| Duzenli,2004 | 2 | Caucasian | Hemorrhagic stroke | 41 | 126 | 4 | 23 |
| Ganaie,2020 | 1 | Asian | Hemorrhagic stroke | 68 | 108 | 5 | 7 |
| Martini,2012 | 2 | Caucasian | Intracerebral Hemorrhage | 597 | 1548 | 97 | 190 |
| Seifert,2006 | 2 | Caucasian | Lobar and Non lobar ICH | 193 | 280 | 22 | 37 |
| Zhang,2012 | 1 | Asian | Intracerebral Hemorrhage | 180 | 180 | 18 | 20 |
| Garcia,1999 | 2 | Caucasian | Lobar ICH | 24 | 24 | 1 | 1 |
| Garcia,1999 | 2 | Caucasian | Deep ICH | 24 | 24 | 3 | 1 |
| Kokubo, 2000 | 1 | Asian | ICH | 84 | 1126 | 6 | 73 |
| Maccarron, 1998 | 2 | Caucasian | SAH | 96 | 406 | 8 | 45 |
| Maccarron, 1998 | 2 | Caucasian | Deep Hemorrhage | 71 | 406 | 7 | 45 |
| Maccarron, 1998 | 2 | Caucasian | CAA Hemorrhage | 40 | 406 | 45 | 9 |
| Tasdemir,2007 | 2 | Caucasian | PICH | 35 | 30 | 2 | 1 |
| Woo,2013 | 2 | Caucasian | Lobar ICH | 204 | 508 | 42 | 59 |
| Woo,2013 | 2 | Caucasian | Non Lobar ICH | 354 | 936 | 49 | 131 |
| Jiang, 2024 | 1 | Asian | ICH | 153 | 60 | 27 | 13 |

**Table 4: Association between APOE ε2/ε4 genotype and the risk of hemorrhagic stroke**

| **Study Author and Year** | **Ethnicity** | **Population** | **Type of Hemorrhage** | **Cases** | **Controls** | **APOE2.4_Cases** | **APOE2.4_Controls** |
| --- | --- | --- | --- | --- | --- | --- | --- |
| Atadzhanov, 2013 | 3 | African | Hemorrhagic stroke | 18 | 116 | 4 | 7 |
| Catto, 2000 | 2 | Caucasian | PICH | 60 | 289 | 2 | 7 |
| Chowdhury, 2000 | 1 | Asian | Cerebral Hemorrhage | 80 | 190 | 0 | 1 |
| Das,2016 | 1 | Asian | Hemorrhagic stroke | 250 | 620 | 19 | 4 |
| Duzenli,2004 | 2 | Caucasian | Hemorrhagic stroke | 41 | 126 | 0 | 2 |
| Ganaie,2020 | 1 | Asian | Hemorrhagic stroke | 68 | 108 | 5 | 1 |
| Martini,2012 | 2 | Caucasian | Intracerebral Hemorrhage | 597 | 1548 | 28 | 37 |
| Seifert,2006 | 2 | Caucasian | Lobar and Non lobar ICH | 193 | 280 | 4 | 6 |
| Zhang,2012 | 1 | Asian | Intracerebral Hemorrhage | 180 | 180 | 6 | 4 |
| Kokubo, 2000 | 1 | Asian | ICH | 84 | 1126 | 2 | 8 |
| Maccarron, 1998 | 2 | Caucasian | SAH | 96 | 406 | 5 | 13 |
| Maccarron, 1998 | 2 | Caucasian | Deep Hemorrhage | 71 | 406 | 3 | 13 |
| Maccarron, 1998 | 2 | Caucasian | CAA Hemorrhage | 40 | 406 | 4 | 13 |
| Misra,2013 | 1 | Asian | RICH | 33 | 188 | 6 | 0 |
| Tasdemir,2007 | 2 | Caucasian | PICH | 35 | 30 | 1 | 1 |
| Woo,2013 | 2 | Caucasian | Lobar ICH | 204 | 508 | 17 | 15 |
| Woo,2013 | 2 | Caucasian | Non Lobar ICH | 354 | 936 | 10 | 22 |
| Woo,2002 | 2 | Caucasian | Lobar ICH | 67 | 131 | 39 | 49 |
| Woo,2002 | 2 | Caucasian | Non Lobar ICH | 121 | 235 | 50 | 102 |
| Hostettler, 2022 | 2 | Caucasian | ICH | 907 | 2636 | 32 | 67 |
| Jiang, 2024 | 1 | Asian | ICH | 153 | 60 | 16 | 7 |

**Table 5: Association between APOE ε3/ε4 genotype and the risk of hemorrhagic stroke**

| **Study Author and Year** | **Ethnicity** | **Population** | **Type of Hemorrhage** | **Cases** | **Controls** | **APOE2.4_Cases** | **APOE2.4_Controls** |
| --- | --- | --- | --- | --- | --- | --- | --- |
| Atadzhanov, 2013 | 3 | African | Hemorrhagic stroke | 18 | 116 | 4 | 7 |
| Catto, 2000 | 2 | Caucasian | PICH | 60 | 289 | 2 | 7 |
| Chowdhury, 2000 | 1 | Asian | Cerebral Hemorrhage | 80 | 190 | 0 | 1 |
| Das,2016 | 1 | Asian | Hemorrhagic stroke | 250 | 620 | 19 | 4 |
| Duzenli,2004 | 2 | Caucasian | Hemorrhagic stroke | 41 | 126 | 0 | 2 |
| Ganaie,2020 | 1 | Asian | Hemorrhagic stroke | 68 | 108 | 5 | 1 |
| Martini,2012 | 2 | Caucasian | Intracerebral Hemorrhage | 597 | 1548 | 28 | 37 |
| Seifert,2006 | 2 | Caucasian | Lobar and Non lobar ICH | 193 | 280 | 4 | 6 |
| Zhang,2012 | 1 | Asian | Intracerebral Hemorrhage | 180 | 180 | 6 | 4 |
| Kokubo, 2000 | 1 | Asian | ICH | 84 | 1126 | 2 | 8 |
| Maccarron, 1998 | 2 | Caucasian | SAH | 96 | 406 | 5 | 13 |
| Maccarron, 1998 | 2 | Caucasian | Deep Hemorrhage | 71 | 406 | 3 | 13 |
| Maccarron, 1998 | 2 | Caucasian | CAA Hemorrhage | 40 | 406 | 4 | 13 |
| Misra,2013 | 1 | Asian | RICH | 33 | 188 | 6 | 0 |
| Tasdemir,2007 | 2 | Caucasian | PICH | 35 | 30 | 1 | 1 |
| Woo,2013 | 2 | Caucasian | Lobar ICH | 204 | 508 | 17 | 15 |
| Woo,2013 | 2 | Caucasian | Non Lobar ICH | 354 | 936 | 10 | 22 |
| Woo,2002 | 2 | Caucasian | Lobar ICH | 67 | 131 | 39 | 49 |
| Woo,2002 | 2 | Caucasian | Non Lobar ICH | 121 | 235 | 50 | 102 |
| Hostettler, 2022 | 2 | Caucasian | ICH | 907 | 2636 | 32 | 67 |
| Jiang, 2024 | 1 | Asian | ICH | 153 | 60 | 16 | 7 |

**Table 6: Association between the APOE ε2 allele and the risk of hemorrhagic stroke**

| **Study Author and Year** | **Ethnicity** | **Population** | **Hemorrhage** | **Cases** | **Controls** | **APOE2_Cases** | **APOE2_Controls** |
| --- | --- | --- | --- | --- | --- | --- | --- |
| Atadzhanov, 2013 | 3 | African | Hemorrhagic stroke | 18 | 116 | 4 | 32 |
| Chowdhury, 2000 | 1 | Asian | Hemorrhagic stroke | 80 | 190 | 1 | 6 |
| Das,2016 | 1 | Asian | Hemorrhagic stroke | 250 | 620 | 32 | 64 |
| Duzenli,2004 | 2 | Caucasian | Hemorrhagic stroke | 41 | 126 | 8 | 29 |
| Ganaie,2020 | 1 | Asian | Hemorrhagic stroke | 68 | 108 | 12 | 8 |
| Zhang,2012 | 1 | Asian | Hemorrhagic stroke | 180 | 180 | 26 | 26 |
| Biffi, 2010, GOCHA | 2 | Caucasian | Lobar ICH | 398 | 555 | 44 | 39 |
| Biffi, 2010, GOCHA | 2 | Caucasian | Deep ICH | 312 | 555 | 22 | 39 |
| Biffi, 2010, GERFHS | 2 | Caucasian | Lobar ICH | 203 | 1304 | 30 | 130 |
| Biffi, 2010, GERFHS | 2 | Caucasian | Deep ICH | 337 | 1304 | 34 | 130 |
| Biffi, 2010, JUHSS | 2 | Caucasian | Lobar ICH | 102 | 429 | 13 | 34 |
| Biffi, 2010, JUHSS | 2 | Caucasian | Deep ICH | 130 | 429 | 12 | 34 |
| Biffi, 2010, MUG-ICH | 2 | Caucasian | Lobar ICH | 77 | 1023 | 7 | 72 |
| Biffi, 2010, MUG-ICH | 2 | Caucasian | Deep ICH | 114 | 1023 | 8 | 72 |
| Biffi, 2010, HM-ICH | 2 | Caucasian | Lobar ICH | 66 | 185 | 7 | 15 |
| Biffi, 2010, HM-ICH | 2 | Caucasian | Deep ICH | 103 | 185 | 9 | 15 |
| Biffi, 2010, LUHSS | 2 | Caucasian | Lobar ICH | 42 | 161 | 5 | 14 |
| Biffi, 2010, LUHSS | 2 | Caucasian | Deep ICH | 89 | 161 | 8 | 14 |
| Biffi, 2010, VHH-ICH | 2 | Caucasian | Lobar ICH | 43 | 87 | 4 | 7 |
| Biffi, 2010, US-AA | 2 | Caucasian | Lobar ICH | 63 | 297 | 9 | 30 |
| Biffi, 2010, US-AA | 2 | Caucasian | Deep ICH | 110 | 297 | 13 | 30 |
| Garcia,1999 | 2 | Caucasian | Lobar ICH | 24 | 24 | 1 | 1 |
| Garcia,1999 | 2 | Caucasian | Deep ICH | 24 | 24 | 2 | 1 |
| Greenberg, 1996 | 2 | Caucasian | Mixed ICH | 12 | 3798 | 1 | 342 |
| Greenberg, 1996 | 2 | Caucasian | Deep ICH | 36 | 3798 | 2 | 342 |
| Kokubo, 2000 | 2 | Caucasian | Hemorrhagic stroke | 84 | 1126 | 14 | 103 |
| Misra,2013 | 1 | Asian | RICH | 33 | 188 | 1 | 15 |
| Misra,2013 | 1 | Asian | NRICH | 101 | 188 | 7 | 15 |
| Nakata,1997 | 1 | Asian | Hemorrhagic stroke | 38 | 38 | 3 | 2 |
| Sawyer.2018 | 2 | Caucasian | Hemorrhagic stroke | 401 | 979 | 93 | 171 |
| Sawyer.2018 | 4 | Hispanic | Hemorrhagic stroke | 269 | 795 | 24 | 69 |
| Sawyer.2018 | 3 | African | Hemorrhagic stroke | 237 | 886 | 47 | 170 |
| Woo,2005 | 5 | Mixed | Lobar ICH | 107 | 107 | 21 | 28 |
| Woo,2002 | 2 | Caucasian | Lobar ICH | 67 | 131 | 17 | 22 |
| Woo,2002 | 2 | Caucasian | Non Lobar ICH | 121 | 235 | 22 | 42 |
| Hostettler, 2022 | 2 | Caucasian | ICH | 907 | 2636 | 188 | 394 |

**Table 7: Association between the APOE ε4 allele and the risk of hemorrhagic stroke**

| **Study Author and Year** | **Ethnicity** | **Population** | **Type of Hemorrhage** | **Cases** | **Controls** | **APOE4_Cases** | **APOE4_Controls** |
| --- | --- | --- | --- | --- | --- | --- | --- |
| Atadzhanov, 2013 | 3 | African | Hemorrhagic stroke | 18 | 116 | 13 | 62 |
| Chowdhury, 2000 | 1 | Asian | Cerebral Hemorrhage | 80 | 190 | 6 | 17 |
| Das,2016 | 1 | Asian | Hemorrhagic stroke | 250 | 620 | 75 | 141 |
| Duzenli,2004 | 2 | Caucasian | Hemorrhagic stroke | 41 | 126 | 3 | 22 |
| Ganaie,2020 | 1 | Asian | Hemorrhagic stroke | 68 | 108 | 15 | 17 |
| Zhang,2012 | 1 | Asian | ICH | 180 | 180 | 54 | 20 |
| Biffi, 2010, GOCHA | 2 | Caucasian | Lobar ICH | 398 | 555 | 84 | 67 |
| Biffi, 2010, GOCHA | 2 | Caucasian | Deep ICH | 312 | 555 | 47 | 67 |
| Biffi, 2010, GERFHS | 2 | Caucasian | Lobar ICH | 203 | 1304 | 43 | 196 |
| Biffi, 2010, GERFHS | 2 | Caucasian | Deep ICH | 337 | 1304 | 54 | 196 |
| Biffi, 2010, JUHSS | 2 | Caucasian | Lobar ICH | 102 | 429 | 13 | 34 |
| Biffi, 2010, JUHSS | 2 | Caucasian | Deep ICH | 130 | 429 | 14 | 34 |
| Biffi, 2010, MUG-ICH | 2 | Caucasian | Lobar ICH | 77 | 1023 | 10 | 102 |
| Biffi, 2010, MUG-ICH | 2 | Caucasian | Deep ICH | 114 | 1023 | 13 | 102 |
| Biffi, 2010, HM-ICH | 2 | Caucasian | Lobar ICH | 66 | 185 | 9 | 17 |
| Biffi, 2010, HM-ICH | 2 | Caucasian | Deep ICH | 103 | 185 | 11 | 17 |
| Biffi, 2010, LUHSS | 2 | Caucasian | Lobar ICH | 42 | 161 | 8 | 26 |
| Biffi, 2010, LUHSS | 2 | Caucasian | Deep ICH | 89 | 161 | 16 | 26 |
| Biffi, 2010, VHH-ICH | 2 | Caucasian | Lobar ICH | 43 | 87 | 5 | 8 |
| Biffi, 2010, US-AA | 2 | Caucasian | Lobar ICH | 63 | 297 | 15 | 56 |
| Biffi, 2010, US-AA | 2 | Caucasian | Deep ICH | 110 | 297 | 22 | 56 |
| Garcia,1999 | 2 | Caucasian | Lobar ICH | 24 | 24 | 3 | 2 |
| Garcia,1999 | 2 | Caucasian | Deep ICH | 24 | 24 | 1 | 2 |
| Greenberg, 1996 | 2 | Caucasian | Mixed ICH | 12 | 3798 | 1 | 532 |
| Greenberg, 1996 | 2 | Caucasian | Deep ICH | 36 | 3798 | 5 | 532 |
| Kokubo, 2000 | 2 | Caucasian | ICH | 84 | 1126 | 23 | 236 |
| Misra,2013 | 1 | Asian | RICH | 33 | 188 | 40 | 18 |
| Misra,2013 | 1 | Asian | NRICH | 101 | 188 | 12 | 18 |
| Nakata,1997 | 1 | Asian | Hemorrhagic stroke | 38 | 38 | 3 | 3 |
| Sawyer.2018 | 2 | Caucasian | White ICH | 401 | 979 | 150 | 234 |
| Sawyer.2018 | 4 | Hispanic | Hiispanic ICH | 269 | 795 | 71 | 167 |
| Sawyer.2018 | 3 | African | Black ICH | 237 | 886 | 89 | 332 |
| Woo,2005 | 5 | Mixed | Lobar ICH | 102 | 187 | 39 | 48 |
| Woo,2002 | 2 | Caucasian | Lobar ICH | 67 | 131 | 24 | 30 |
| Woo,2002 | 2 | Caucasian | Non Lobar ICH | 121 | 235 | 29 | 66 |
| Hostettler, 2022 | 2 | Caucasian | ICH | 907 | 2636 | 255 | 789 |
| Jiang, 2024 | 1 | Asian | ICH | 153 | 60 | 40 | 18 |
